# Coherent Markov Random Field-Based Unreliable DSM Areas Segmentation and Hierarchical Adaptive Surface Fitting for InSAR DEM Reconstruction

**DOI:** 10.3390/s20051414

**Published:** 2020-03-04

**Authors:** Qian Qian, Bingnan Wang, Xiaoning Hu, Maosheng Xiang

**Affiliations:** 1 National Key Laboratory of Microwave Imaging Technology, Aerospace Information Research Institute, Chinese Academy of Sciences, Beijing 100190, China; huxiaoning16@mails.ucas.ac.cn (X.H.); xms_iecas@163.com (M.X.); 2 School of Electronic, Electrical and Communication Engineering, University of Chinese Academy of Sciences, Beijing 100190, China

**Keywords:** coherence coefficient, DEM, DSM, hierarchical adaptive surface fitting, InSAR, markov random field, residue

## Abstract

A digital elevation model (DEM) can be obtained by removing ground objects, such as buildings, in a digital surface model (DSM) generated by the interferometric synthetic aperture radar (InSAR) system. However, the imaging mechanism will cause unreliable DSM areas such as layover and shadow in the building areas, which seriously affect the elevation accuracy of the DEM generated from the DSM. Driven by above problem, this paper proposed a novel DEM reconstruction method. Coherent Markov random field (CMRF) was first used to segment unreliable DSM areas. With the help of coherence coefficients and residue information provided by the InSAR system, CMRF has shown better segmentation results than traditional traditional Markov random field (MRF) which only use fixed parameters to determine the neighborhood energy. Based on segmentation results, the hierarchical adaptive surface fitting (with gradually changing the grid size and adaptive threshold) was set up to locate the non-ground points. The adaptive surface fitting was superior to the surface fitting-based method with fixed grid size and threshold of height differences. Finally, interpolation based on an inverse distance weighted (IDW) algorithm combining coherence coefficient was performed to reconstruct a DEM. The airborne InSAR data from the Institute of Electronics, Chinese Academy of Sciences has been researched, and the experimental results show that our method can filter out buildings and identify natural terrain effectively while retaining most of the terrain features.

## 1. Introduction

An interferometric synthetic aperture radar (InSAR) has the ability of acquiring a large-area and high-precision digital surface model (DSM) in all-times and all weather. The information of a digital elevation model (DEM) is required for many applications, therefore it is necessary to reconstruct a DEM from a DSM by removing the above-ground objects such as buildings. The DEM reconstruction is involved in photogrammetry [1,2], laser detection and ranging (LiDAR) [3,4,5,6], or InSAR [7,8,9,10]. Many methods have been proposed in this subject especially in the field of LiDAR [6], however reconstruction research based on InSARs is relatively rare. The main reason is that the accuracy of an InSAR DSM is lower than that of LiDAR due to the unique side looking imaging mechanism of synthetic aperture radar (SAR). For example, in an InSAR DSM, there can be a lot of layover and shadow areas in the building scene and the interferometric phase inversion of these areas are not reliable, which may generate a lot of points with incorrect extreme elevations in an InSAR DSM. Therefore, the reconstruction of an InSAR DEM is more challenging than that of LiDAR data.

Wang and Mercer [7] proposed an InSAR DEM reconstruction method based on image pyramid. Each level needs to be reconstructed in this algorithm, thus the error in the middle level will affect the next level, which is prone to error accumulation. Jiang [8] combined the slope information and the image pyramid method to filter non-ground points by calculating the slope between the candidate points. Zhang and Tao [9] proposed a surface-fitting-based method of an InSAR DEM reconstruction. The DEM is generated from InSAR DSM by extracting candidate ground points in a fixed-size grid, adjusting points with a distance of more than the given threshold from fitted surface, and and using ground points for interpolatio. These methods assume that the point with the minimum elevation in the fixed-size grid is the ground point, without considering the unreliable DSM points with the large spike noise belonging to layovers and shadows in the InSAR building areas. When the local minimum points fall into these unreliable DSM areas, extreme points are selected as the ground points, causing significant errors in the DEM reconstruction. Therefore, to avoid the adverse effects of these areas on ground points selection, it would make sense to segment the unreliable DSM area before selecting the ground point. At the same time, the selection of grid size and threshold in surface fitting may also significantly affect the reconstruction of the DEM. When the grid size is too large, some ground details will be lost, and the terrain will be smoothed. When the grid size is too small, the local minimum point will fall into the building, resulting in reduced DEM reconstruction accuracy.

Unreliable DSM areas mainly include the layover and shadow in a building scene, which can be segmented by the intensity of pixel gray because of their different brightness in SAR images. Due to the existence of speckle noise and complex texture characteristics of ground objects, the segmentation results are not satisfactory in the general image segmentation algorithm. To improve segmentation performance, the spatial relationship is usually considered. Markov random field (MRF) is recognized in the field of image segmentation due to its ability to utilize spatial context information [11], and it has been widely applied in SAR image segmentation [12,13,14]. In a traditional MRF, the ability of the neighborhood energy to describe the spatial correlation is insufficient, and the fixed parameter causes the neighborhood pixels to have the same impact on the central pixel. Moreover, the context information is not fully utilized [15,16], therefore the segmentation result is prone to misclassification points. In this paper, considering the potential of interferometric information and the coherence coefficient and residue information are incorporated into the traditional MRF model for improving segmentation performance.

Based on the above discussion, this paper proposed a DEM reconstruction method based on unreliable DSM area segmentation and hierarchical adaptive surface fitting. The contributions of this paper can be summarized as follows:

(1) In order to avoid the influence of the extreme points in the unreliable DSM areas when performing DEM reconstruction, segmentation based on the intensity of pixel gray levels in the InSAR amplitude image (which is helpful for the selection of ground points) was firstly used to identify the unreliable DSM areas for improving the performance of the subsequent DEM reconstruction.
(2) In order to improve the segmentation performance, we considered the potential of InSAR data information, such that this paper combined the coherence coefficient and residue information of interferometric phase with the neighborhood energy of the MRF, and the full use of contextual relationship was achieved by using the interferometric information between neighboring pixels.
(3) In the general surface fitting-based method, the fixed grid size and threshold will affect the filtering accuracy. Therefore, a new idea of progressively reducing the grid size and setting the adaptive threshold is proposed. It can realize the step-by-step filtering of ground points and the preservation of terrain detail information. At the same time, inverse distance weighted (IDW) interpolation with coherence coefficient is performed for completing the reconstruction of the DEM.

The rest of the paper is organized as follows. In Section 2, details of the proposed method are described. The experimental results and discussion are in Section 3, and Section 4 is the conclusions.

## 2. Proposed Method

### 2.1. Unreliable DSM Areas Segmentation with Coherent Markov Random Field (CMRF) Method

#### 2.1.1. Image Segmentation Based on a MRF Model

A MRF model regards an image as a points set S, and the segmentation label X is a random field corresponding to S. The spatial relationship between neighboring pixels is constructed by defining neighborhood cliques $\eta =\{\eta_{ij}:\;(i,j)\in S,\eta_{ij}\in S\}$. According to Bayesian theory, we need to find the estimate of segmentation label $X_{MAP}$ that maximizes the posterior probability distribution:

(1) $${\hat{X}}_{MAP}=\arg\max P(X|Y)=\arg\max\frac{P(Y|X)P(X)}{P(Y)}=\arg\max P(Y|X)P(X)$$

where X is the segmentation label, and Y is the observation image. According to the equivalence of MRF and Gibbs Random Field (GRF), which can be proved by the Hammersley-Clifford theorem and the Gibbs theorem, the posterior probability distribution can be represented as:

(2) $$P(X=x|Y=y)=Z^{-1}\exp(-U(x|y))$$

where U is the energy function; and Z denotes the normalizing constant. From Equation (2), it can be seen that maximizing the posterior probability P(X|Y) means minimizing energy function U(x|y). Moreover U(x|y) which is called posterior energy in this letter can be decomposed into Equation (3)

(3) $$\begin{array}{cc} U(x|y) & =U(y|x)+U(x)\\ & =-\sum_{s}\ln p(y_{s}|x_{s})+\sum_{c\in V_{s}}V_{c}(x) \end{array}$$

where $V_{s}$ is a set of all neighborhood cliques; U(y|x) denotes the likelihood energy which represents the contribution of the pixel itself to the energy; and U(x) denotes the neighborhood energy. $V_{c}(x)$ is expressed as Equation (4) [17]:

(4) $$V_{c}(x)=\left\{ \begin{array}{ll} 0 & x_{i}=x_{j}\\ \beta & x_{i}\neq x_{j} \end{array}\right.$$

where $x_{i}$ is the segmentation label of pixel i; $x_{j}$ is the segmentation label of pixel j which is neighboring pixel of i; and β is a parameter to control the contribution between U(y|x) and U(x), which is usually determined by experience.

As shown in Equation (3), the likelihood energy is related to the likelihood function of pixels. According to the imaging structure and pixel gray of the building scene in the SAR image, the following three classes are determined, and the unreliable DSM areas include the layover and shadow areas.

(1) Layover areas: The characteristics of this area are scattered signals of targets at different positions overlapping at the same distance resolution unit, causing high brightness in the SAR image.
(2) Shadow areas: This area is characterized by an extremely low backscattered signal strength, which is caused by steep terrain or occlusion by towering targets.
(3) Background areas: The other areas which don’t belong to the layover or shadow in the scene are grouped into the background, which mainly includes roofs, trees, and bare ground.

A Fisher distribution model is used to describe the probability distribution of building scenes in high-resolution SAR images by Tison [18], and it can be described as follows:

(5) $$p_{Fisher}(u)=\frac{\Gamma (L+M)}{\Gamma (L)\Gamma (M)}\frac{L}{M\mu }\frac{{\left(\frac{L}{M\mu }u\right)}^{L-1}}{{\left(1+\frac{L}{M\mu }u\right)}^{L+M}},L>0,M>0$$

where L and M represent the shape parameters; μ denotes the weight parameter; and Γ is the Gamma function.

After selecting areas of different classes defined above as the supervising information, we can estimate the parameters as follows:

(6) $$M=\frac{4R_{1}-3R_{2}-1}{2R_{1}-R_{2}-1}$$

(7) $$L=\frac{2(R_{1}-R_{2})}{-R_{1}+2R_{2}-R_{1}R_{2}}$$

(8) $$\mu =m_{1}\frac{2(R_{1}-R_{2})}{4R_{1}-3R_{2}-1}$$

where $R_{1}=m_{2}/(m_{1}*m_{1})$, $R_{2}=m_{3}/(m_{1}*m_{2})$, and $m_{1}$, $m_{2}$, $m_{3}$ are the statistical histogram central moments of corresponding orders. 

Therefore, according to the estimated Fisher probability distribution corresponding to Equation (5) and the neighborhood energy shown in Equation (4), the class label can be obtained by the following formula:

(9) $$\begin{array}{cc} \hat{X} & =\arg\min U(x|y)\\ & =\arg\min(-\sum_{s}\ln p(y_{s}|x_{s})+\sum_{c\in V_{s}}V_{c}(x)) \end{array}$$

These class labels are firstly obtained by the initial segmentation, and then the labels are updated iteratively. The neighborhood energy is related to the class labels of the neighboring pixels, and the likelihood energy is determined by the probability distribution function of the pixel values. The pixel value and neighboring label are used to calculate the posterior energy of a single pixel, and the label with the minimum energy value is used as the segmentation result. Finally, iterative solution is performed until the energy is stable.

#### 2.1.2. CMRF Segmentation

In the traditional MRF model, when the center pixel label and the neighborhood pixel label are the same, the neighborhood energy is a certain value, and when the labels are different, it is zero. This results in the adjacent pixels having the same effect on the center pixel [15], therefore it cannot fully utilized the contextual information. Driven by this problem, this paper redefined the neighborhood energy model of MRF based on the coherence coefficient and residue information to make full use of the contextual interferometric information. 

The coherence coefficient is used to evaluate the quality of the InSAR interferogram, which is defined as follows [19]:

(10) $$\gamma =\frac{\left|E\left[s_{1}s_{2}{}^{*}\right]\right|}{\sqrt{E\left[{\left|s_{1}\right|}^{2}\right]E\left[{\left|s_{2}\right|}^{2}\right]}}$$

where $s_{1}$ and $s_{2}$ are the interferometric complex image pair; and E represents mathematical expectation. The interferometric coherence is an elemental parameter for InSAR applications, which is estimated by comparing the radar echo across several nearby radar images pixels [20]. The related coherent change detection (CCD) [21], maximum-likelihood (ML) CCD [22], and ML-polarimetric InSAR-CCD (ML-PolInSAR-CCD) [23] are important applications of satellite earth observation. The coherence coefficient is related to the characteristics of the scatterers. For example, pixels which belong to shadow area tend to have low a coherence coefficient because the scattering signal in these areas is dominated by noise, while the coherence coefficient in other areas is usually higher than shadow. This property can be used to distinguish different classes [24]. Meanwhile, the coherence coefficient usually shows consistency and uniformity in areas with pixels belonging to the same category, which can be used to further improve the performance of image segmentation. This paper defines a coherence coefficient distance that measures the difference in coherence between the central pixel and the neighboring pixels, and it is expressed as follows:

(11) $$D=\left|\gamma_{i}-\gamma_{j}\right|$$

where $\gamma_{i}$ is the coherence coefficient of the pixel i; and $\gamma_{j}$ is the coherence coefficient of pixel j, which is the neighboring pixel of i.

Furthermore, the residue information of the interferometric phase is also helpful for SAR image segmentation. Under ideal conditions, the absolute value of the phase gradient should be less than π. However, due to the existence of low scattering areas such as shadow, smooth roads, and water, etc., the absolute value of the wrapped phase gradient may be greater than π. This is called the phase discontinuity point and is known as residue [25]. The residue distribution in the interferometric phase image is obtained according to the following formula:

(12) $$\begin{array}{l} \psi_{1}=W(\varphi_{i,j+1}-\varphi_{i,j})\\ \psi_{2}=W(\varphi_{i+1,j+1}-\varphi_{i,j+1})\\ \psi_{3}=W(\varphi_{i+1,j}-\varphi_{i+1,j+1})\\ \psi_{4}=W(\varphi_{i,j}-\varphi_{i+1,j})\\ R=\psi_{1}+\psi_{2}+\psi_{3}+\psi_{4} \end{array}$$

where $\varphi_{i,j}$ represents the wrapped phase at the pixel (x,y); and W represents the wrapped phase operator. When R>0 it is a positive residue, otherwise it is a negative residue, and R=0 is the normal point. The residues are caused by phase discontinuity in low-scattering areas such as shadows. If both points are residues, they are likely to be divided into shadows, thus residue information can be helpful for segmenting InSAR amplitude images. 

Considering the effects of coherence coefficient and residue information, if the coherence coefficient distance is small and both points are residues, the possibility of being divided into the same class is greater, and vice versa.

**Figure 1:**
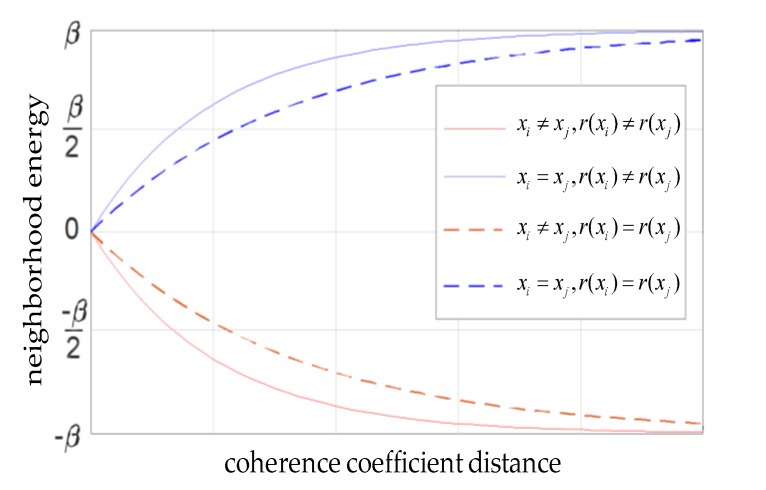
Curve of the neighborhood energy changing with the coherence coefficient distance and residue information.

More specifically, when the class labels are the same between the center pixel and the neighboring pixel, a small coherence coefficient distance should mean low neighborhood energy, which may increase the probability of being identified as the same class for the two pixels. Meanwhile, if the center pixel and the neighboring pixel are both residues, the corresponding neighborhood energy should be lower than the energy that the two points are not both residues, and it is more likely to be classified into the same label. When the class labels are different between the center pixel and the neighboring pixel, the opposite is true. Based on the above analysis, the improved neighborhood energy form is as follows:

(13) $$V_{c-CMRF}(x)=\left\{ \begin{array}{l} \left(1-e^{-\alpha D})\beta \;\;\;\;\;\;x_{i}=x_{j}\;\;\;r(x_{i})\neq r(x_{j}\right)\\ \left(1-e^{-\mu \alpha D})\beta \;\;\;\;x_{i}=x_{j}\;\;\;r(x_{i})\;=\;r(x_{j}\right)\\ \left(e^{-\alpha D}-1)\beta \;\;\;\;\;\;x_{i}\neq x_{j}\;\;\;r(x_{i})\neq r(x_{j}\right)\\ \left(e^{-\mu \alpha D}-1)\beta \;\;\;\;x_{i}\neq x_{j}\;\;\;r(x_{i})\;=\;r(x_{j}\right) \end{array}\right.$$

where α is a constant greater than zero and it is used to control the shape of the curve; μ is the weighting coefficient of the residue information; and $r(x_{i})\;=\;r(x_{j})$ means that both $x_{i}$ and $x_{j}$ are residues, and $r(x_{i})\neq r(x_{j})$ means the opposite. Figure 1 shows a curve of the neighborhood energy as a function of coherence coefficient distance and residue information. 

Therefore, Equation (3) is represented as Equation (14) in the CMRF model:

(14) $$U_{CMRF}(x|y)\;=-\sum_{s}\ln p(y_{s}|x_{s})+\sum_{c\in V_{s}}V_{c-CMRF}(x)$$

Equation (9) is represented as Equation (15):

(15) $$\begin{array}{cc} \hat{X} & =\arg\min U_{CMRF}(x|y)\\ & =\arg\min(-\sum_{s}\ln p(y_{s}|x_{s})+\sum_{c\in V_{s}}V_{c-CMRF}(x)) \end{array}$$

### 2.2. DEM Reconstruction Based on Hierarchical Adaptive Surface Fitting

#### 2.2.1. Reconstruction Method Based on Surface Fitting

After removing the points of the unreliable DSM areas, the lowest points of the grids which don’t belong to the unreliable DSM areas are used for surface fitting to realize DEM reconstruction. Zhang [9] took the local minimum points in a given grid as the candidate ground points, which were further optimized by surface fitting. Assuming that the terrain surface is a complex spatial surface, and it can be approximated by a quadric surface, as shown in the following equation:

(16) $$z=a_{0}+a_{1}x+a_{2}y+a_{3}x^{2}+a_{4}y^{2}+a_{5}xy$$

where z represents the value of DEM; and x,y represent the horizontal and vertical coordinates of the candidate ground points, respectively. According to the least squares method, the parameters of the surface equation can be determined by the following equation:

(17) $$A={\left(M^{T}PM\right)}^{-1}(M^{T}PZ)$$

where $A={\left[a_{0},a_{1},\cdots ,a_{5}\right]}^{T}$, $Z={\left[z_{1},z_{2},\cdots ,z_{n}\right]}^{T}$. M, and P are described as follow:

(18) $$M=\left[\begin{array}{cccccc} 1 & x_{1} & y_{1} & x_{1}{}^{2} & x_{1}y_{1} & y_{1}{}^{2}\\ 1 & x_{2} & y_{2} & x_{2}{}^{2} & x_{2}y_{2} & y_{2}{}^{2}\\ \vdots & \vdots & \vdots & \vdots & \vdots & \vdots \\ 1 & x_{n} & y_{n} & x_{n}{}^{2} & x_{n}y_{n} & y_{n}{}^{2} \end{array}\right]$$

(19) $$P=\left[\begin{array}{cccc} p_{1} & & & 0\\ & p_{2} & & \\ & & \ddots & \\ 0 & & & p_{n} \end{array}\right]$$

where $p_{1},p_{2},\cdots ,p_{n}$ are the weights of the corresponding points. This paper considers that all points have the same effect on surface fitting, therefore $p_{1}=p_{2}\cdots =p_{n}=1$, and n is the number of points used for fitting. If the difference between the actual elevation and the fitted elevation is greater than the given threshold, the point is filtered out; otherwise, the original value remains unchanged. 

**Figure 2:**
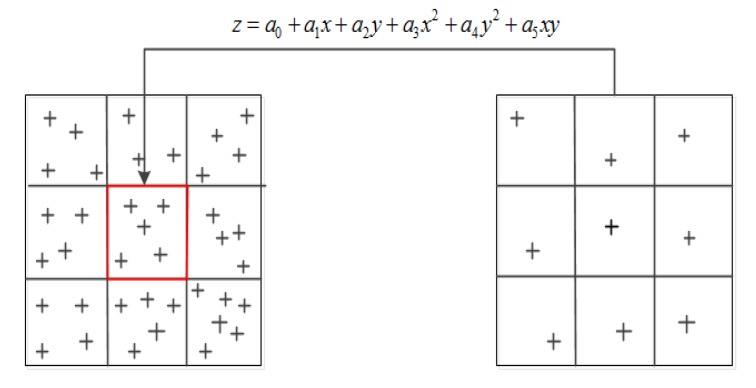
Fitting process with minimum points in neighborhood grids.

Considering the continuity of the terrain, this paper added the neighborhood grids, and the fitting surface of each grid is obtained based on the minimum points which exclude the detected unreliable DSM points of the 3 × 3 neighborhood grids, as shown in Figure 2. The left part represents the original DSM data, and the red grid is surrounded by its 3 × 3 neighborhood grids. The point in each grid in the right part is the local lowest point of the grid, which cannot be the detected DSM unreliable point. These points in the right part are fitted to the surface of the red grid by Equation (16). The surface fitting using the minimum points of the neighborhood grids can maintain the characteristics of the terrain as much as possible.

**Figure 3:**
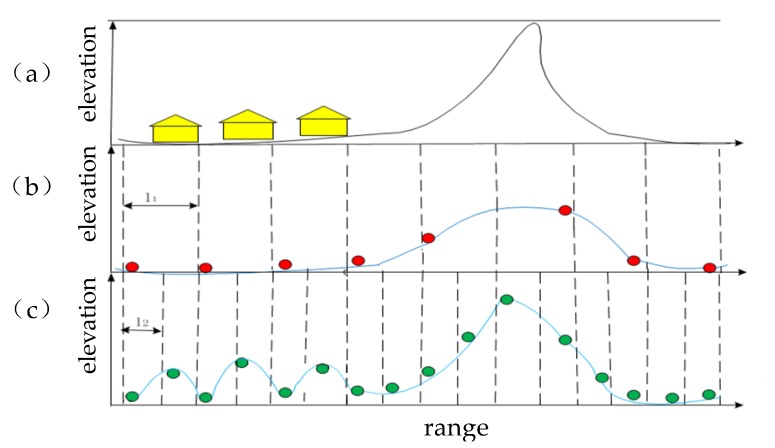
Ground points selection for a steep terrain area with buildings. In part (**a**), buildings and terrain are shown in different colors. In parts (**b**) and (**c**), dashed lines define the grid cells for ground points selection; the red and blue circles represent the lowest points, and the blue lines represent the initial terrain constructed with the lowest points.

In the surface fitting-based method, the choice of grid size is important. As shown in Figure 3, when the grid size is set to a large value such as $l_{1}$, the lowest point will not fall near the ridge, thus it is difficult to completely retain the true terrain at the ridge during subsequent surface fitting. When the grid size is set to a small value such as $l_{2}$, the lowest point will fall on the roof of the building, and the fitted terrain will deviate from the real terrain, resulting in incomplete filtering of the buildings. At the same time, the threshold in the filtering process is not changed adaptively, which will affect the reconstruction result.

#### 2.2.2. Hierarchical Adaptive Surface Fitting

In order to solve the above problem, this paper proposed a hierarchical adaptive surface fitting method. Inspired by Zhang [9], the performance of the algorithm is improved by the following process.

**Figure 4:**
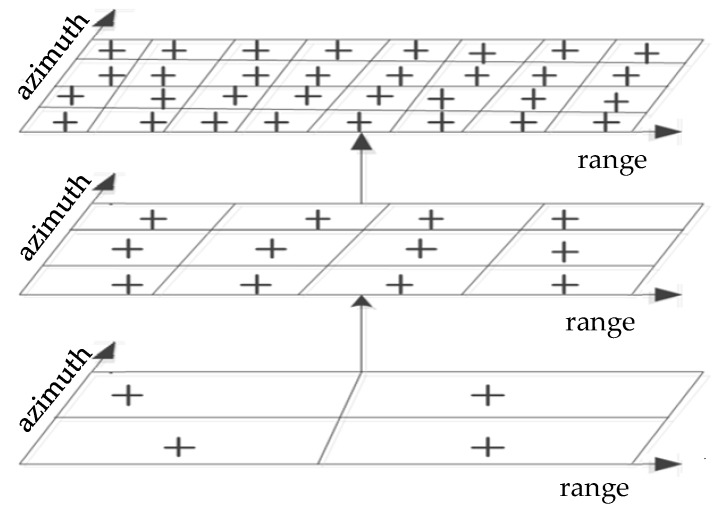
Hierarchical surface fitting with decreasing grid.

**Figure 5:**
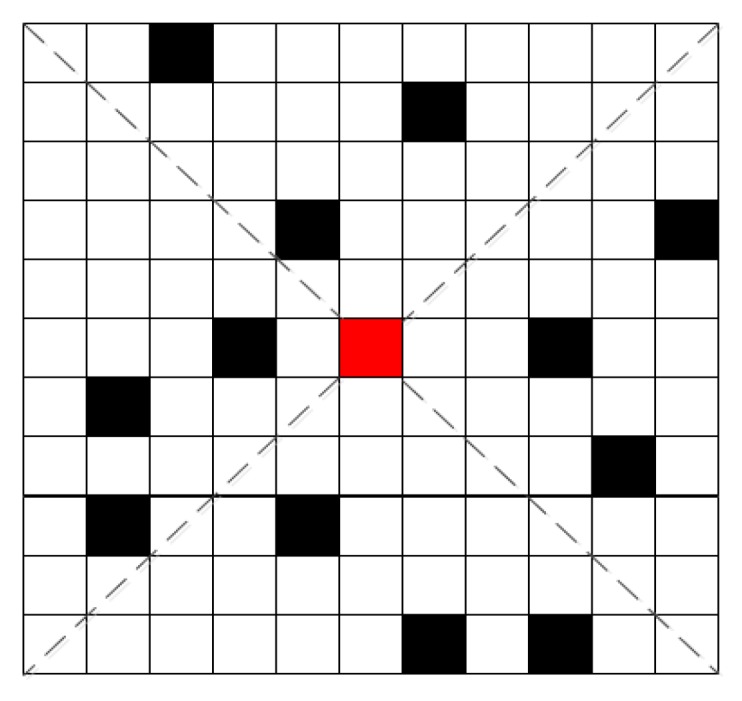
Inverse distance weighted (IDW) algorithm. Black boxes represent ground points, and red boxes represent points to be interpolated.

(1) Hierarchical surface fitting: In the first iteration, the DSM data is first divided evenly by relatively large-sized grids, and then the minimum elevation points in each grid that are not the unreliable DSM areas are used as candidate ground points. The candidate ground points are compared with the surface obtained by fitting the candidate ground points in the 3 × 3 neighborhood grids. If the difference between the elevation of the candidate ground point and the fitted surface is greater than the threshold, the candidate point will be marked as non-ground points. Due to the large mesh size in the first iteration, it cannot represent the true topographic relief well, and the threshold should be set relatively loosely, filtering out buildings with large elevation values. In order to further locate potential non-ground points, we continuously reduce the size of the mesh and repeated the above steps until the mesh size is less than the preset minimum. Figure 4 shows a schematic diagram of the hierarchical surface fitting process.
(2) Determination of adaptive threshold: As mentioned above, considering the influence of grid size and elevation variance, this paper proposed a method for adaptively determining the threshold, which is shown in the Equation (20). The basic idea is that smaller grid size and variance of elevation difference usually correspond to a more reliable fitting result, which means that the threshold should be relatively strict. Conversely, with the increase of grid size and variance, its ability to represent real terrain is weakened, indicating that the fitted terrain has large deviations and the threshold should be relatively loose.
  (20) $$T=\mu_{1}\times l+\mu_{2}\times \sigma^{2}$$
  where l represents the grid size; and $\sigma^{2}$ represents the variance of elevation difference. $\mu_{1}$ and $\mu_{2}$ represent the weights of the grid size and variance of elevation difference, respectively.
(3) Interpolation with Coherence-Coefficient-Based IDW: After the ground points have been acquired by hierarchical surface fitting, the next step is to perform the interpolation with discrete ground points. In this study, the IDW algorithm was selected to interpolate the ground DEM, and it determines the weighting coefficient of ground points based on the distance between the known ground point and the interpolation point. This algorithm searches for ground points within the initial area, and if the number of ground points meets the set threshold, the search is stopped and then the weight of the searched ground points is calculated and interpolation is performed; otherwise the search radius is increased and the search is continued until the condition is satisfied. Figure 5 shows the algorithm execution diagram. When calculating the elevation of the red box, which is the point to be interpolated, search for ground points around it. If the number of black boxes representing the ground points reaches the set threshold, the distance between each ground point and the point to be interpolated is calculated, and then the weight $\omega_{i-IDW}$ is obtained by Equation (21).
  (21) $$\omega_{i-IDW}=\frac{\frac{1}{d_{i}{}^{2}}}{\sum_{n=1}^{N}\frac{1}{d_{n}{}^{2}}}$$
  where $d_{i}$ is the distance between the ground point i and the point to be interpolated; and N is the number of points participating in the calculation. Finally, the product of the weight and the elevation of ground point is summed to obtain the elevation of the point to be interpolated.

Considering the influence of the coherence coefficient, we combine the coherence coefficient and the inverse distance to improve the determination of the weight. The weight $\omega_{i}$ of the ground point i is expressed as follows:

(22) $$\omega_{i}=\frac{\frac{q_{i}}{d_{i}{}^{2}}}{\sum_{n=1}^{N}\frac{q_{n}}{d_{n}{}^{2}}}$$

where $q_{i}$ is the coherence coefficient of ground point i. The elevation of the point to be interpolated is estimated with the weighted sum:

(23) $$h=\sum_{i=1}^{N}\omega_{i}h_{i}$$

where h represents the elevation of the point to be interpolated; and $h_{i}$ represents the elevation of the ground point i.

**Figure 6:**
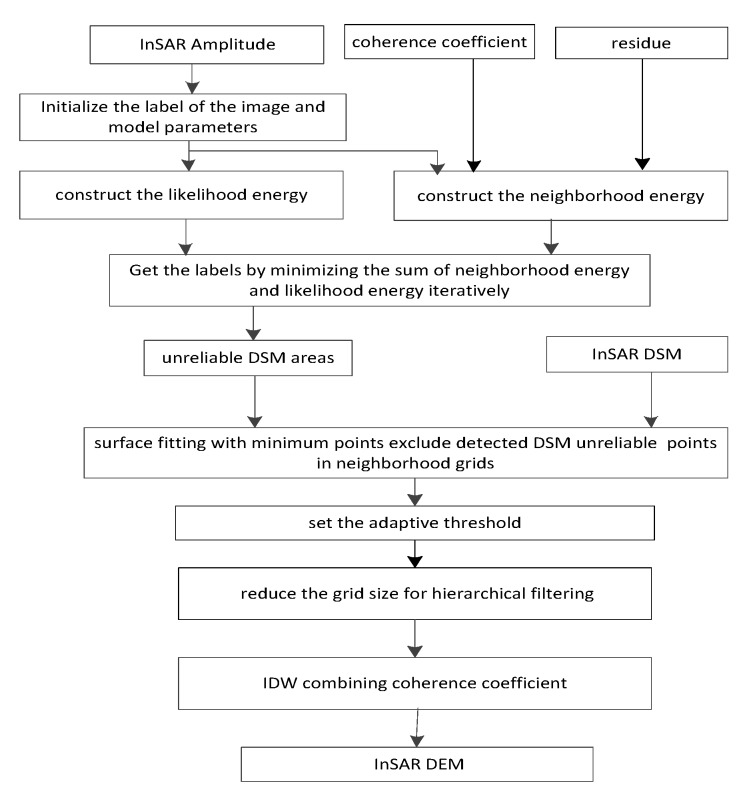
Flowchart of proposed digital elevation model (DEM) reconstruction method.

As mentioned above, a DEM reconstruction method based on unreliable DSM area segmentation and hierarchical adaptive surface fitting was proposed in this method. As shown in Figure 6, in this method, an InSAR amplitude image is segmented initially, and the InSAR coherence coefficient and residue of interferometric phase are plugged into the neighborhood energy of the MRF model. Then we construct the likelihood energy and find the class labels that minimize the sum of the likelihood energy and the neighborhood energy as the segmentation result of the unreliable DSM areas. Next, the DSM is divided by a uniform grid and the minimum points of each neighborhood grids, which do not belong to the unreliable DSM area such as building layover and shadow, are used to fit a quadratic elevation surface. The difference between the true elevation and the fitted elevation is then calculated, and the points that are higher than the designed adaptive threshold are filtered out. Then the grid size changes step-by-step, iteratively filtering out the non-ground points. The surface fitting and filter is iterated in turn until the filter effect is not significantly different, or the filtering is stopped when the max number of iterations are met. Finally, the IDW interpolation combining the coherence coefficient is performed for completing the reconstruction of the DEM. 

## 3. Results

### 3.1. Testing Data

In this paper, the InSAR data used for experimental verification was obtained by the Ku-band frequency modulation continuous wave (FMCW) InSAR system of the Institute of Electronics, Chinese Academy of Sciences in November 2015. The relative flight altitude of this experimental carrier aircraft was 1500 m, the incidence angle was 45 degrees, and the step size of DSM was 0.06 m. The experimental area was located in Jishan County, Yuncheng City, Shanxi Province, and belongws to hilly terrain where the buildings were densely distributed, and the terrain height was between 340 m and 420 m. The laser detection and ranging (LiDAR) bald earth DEM data from the same region was used as the reference DEM. 

**Figure 7:**
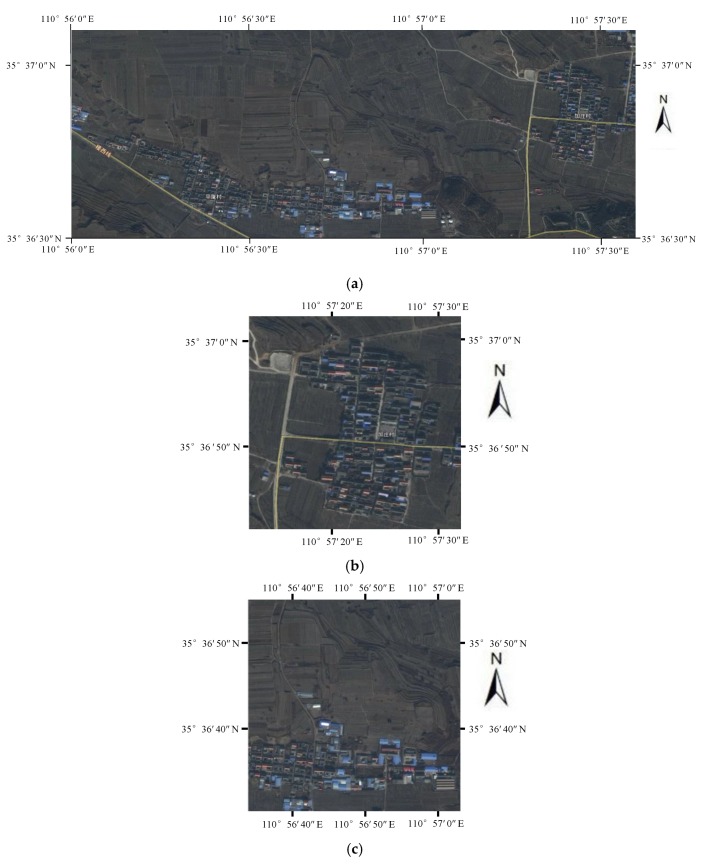

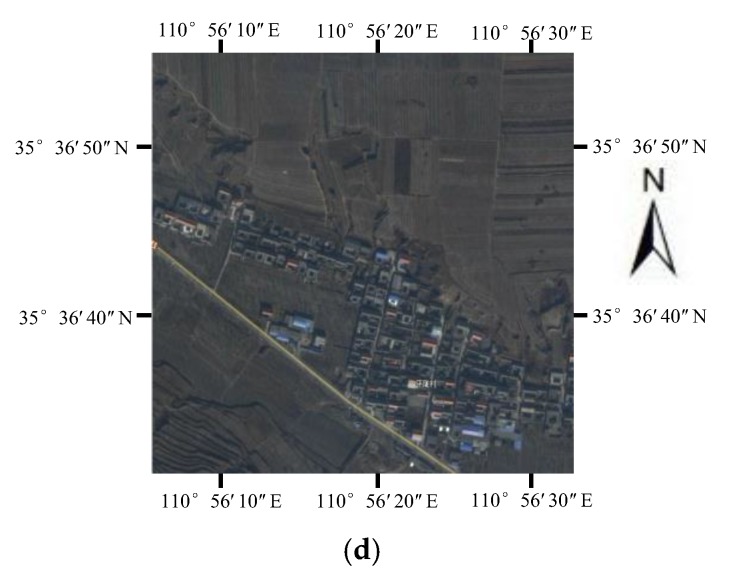
Optical image of experimental and evaluation areas. (**a**) Optical image of experimental areas. (**b**–**d**) Optical image of Site A to B.

In this experimental data, three sites with buildings densely distributed were selected to evaluate the reconstruction results. Figure 7 shows the optical images of experimental areas.

### 3.2. The Segmentation Result of CMRF-Based Unreliable DSM Areas

**Figure 8:**
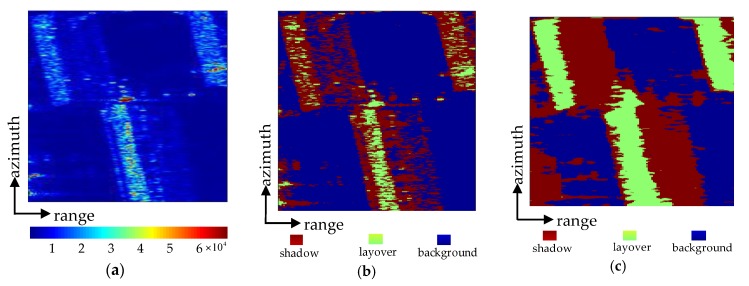
(**a**) The buildings in interferometric synthetic aperture radar (InSAR) amplitude image, and (**b**,**c**) the segmentation results based on traditional Markov random field (MRF) and coherent Markov random field (CMRF), respectively.

According to Equations (6)–(8), the parameters of Fisher distribution were calculated in three areas, and the results are shown in Table 1. Thus, the likelihood energy could be obtained. Then the image was initially segmented, and its neighborhood energy could be calculated according to Equation (4). Finally, we found the class labels that minimize the sum of the likelihood energy and the neighborhood energy. This process needs to be iteratively calculated. An amplitude image of buildings was selected in the test sites for experiments, and the experimental results are shown in Figure 8.

The building scene is shown in Figure 8a. Figure 8b,c are the segmentation results using traditional MRF and CMRF, respectively, where green represents layover and blue represents shadow, and red represents background areas.

It can be seen from Figure 8b that segmentation results generated by traditional MRF contain lots of holes and misclassifications. As shown in Figure 8c, the CMRF method detected most of the unreliable DSM areas and gave a better visual effect. The reason is that the introduction of coherence coefficient and residue can help the classifier make use of the interferometric information and better segment the InSAR amplitude image.

### 3.3. The DEM Reconstruction Result

**Figure 9:**
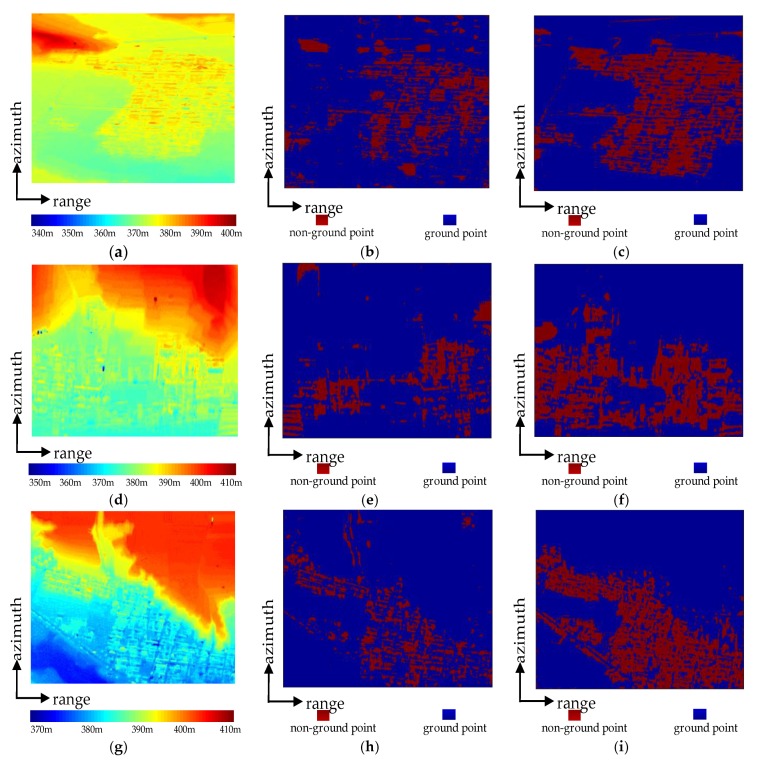
Hierarchical surface fitting results. (**a**), (**d**), and (**g**) are the InSAR digital surface model (DSM); (**b**), (**e**), and (**h**) are the first surface fitting results; and (**c**), (**f**), and (**i**) are the third surface fitting results.

In order to verify the effectiveness of hierarchical surface fitting, Figure 9 shows the first filtering result and the third filtering result. The ground and non-ground points of first filtering results are shown in Figure 9b,e,h, and the third filtering results are shown in Figure 9c,f,i, where blue represents the ground points and red indicates non-ground points. It can be seen from Figure 9b,e,h that some non-ground points are not detected in the first filtering, and some ground points are mistakenly classified as non-ground points, indicating that the grid size and the threshold of first filtering is too large. Therefore some non-ground points have not been filtered out, and the large grid size has lost the terrain detail information, causing some fluctuating ground points to be misidentified as non-ground points. The third fitting had detected more non-ground points than the first fitting, and the number of misjudging points was less, which means that buildings can be filtered out step-by-step while maintaining terrain features, indicating the effectiveness of hierarchical surface fitting.

**Figure 10:**
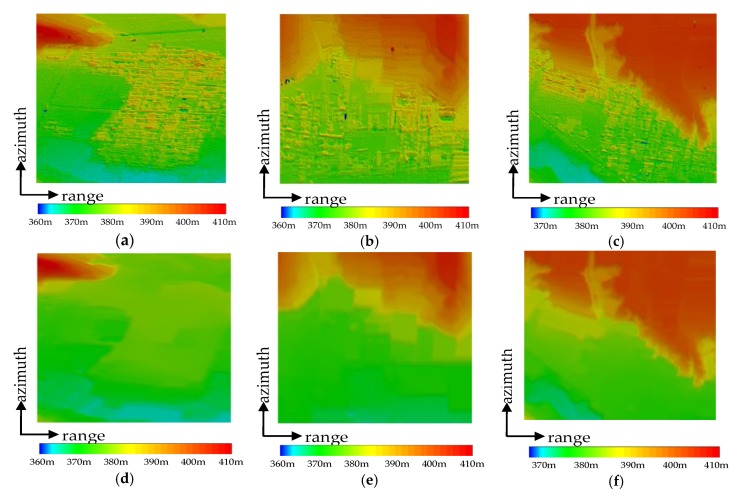

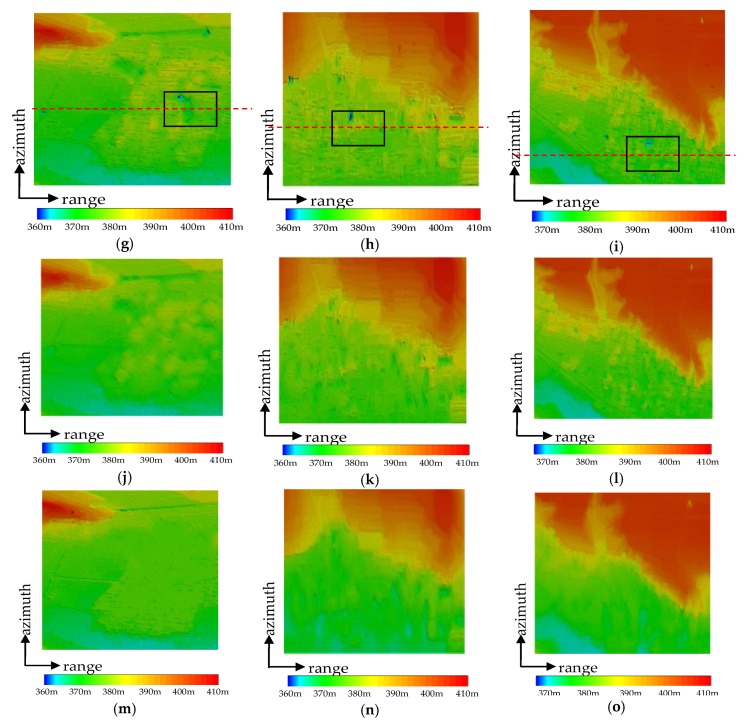
Visual comparison of before and after processing of the InSAR DSM, where (**a**–**c**) are the original DSMs, (**d**–**f**) are the reference DEMs obtained by laser detection and ranging (LiDAR), (**g**–**i**) are the reconstructed DEMs based on surface fitting, (**j**–**l**) are the reconstructed DEMs based on coherent Markov random field (CMRF)+surface fitting, and (**m**–**o**) are the reconstructed DEMs based on proposed method.

**Figure 11:**
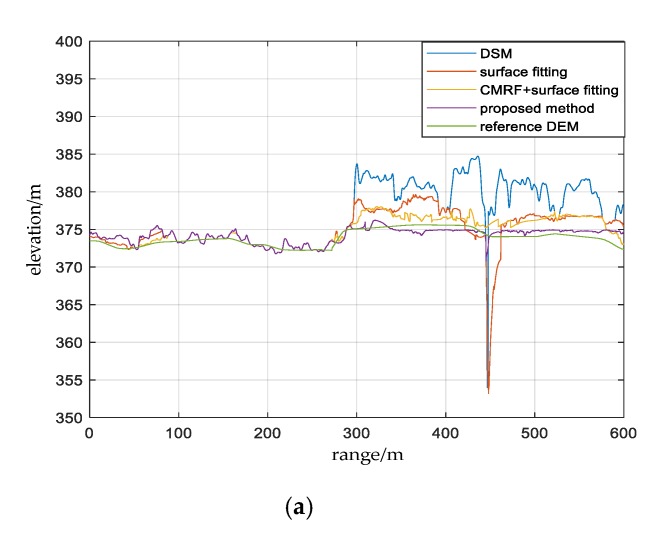

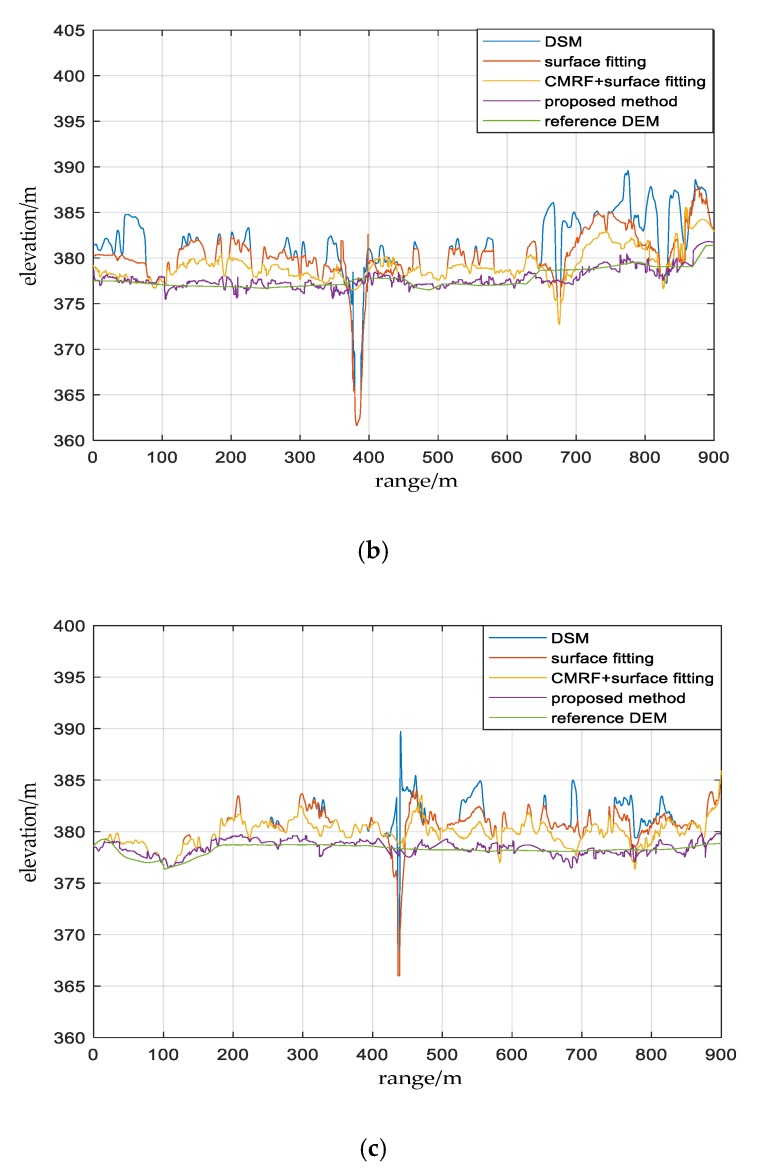
Altimetric profile comparison between the DSM, the reconstructed DEM based on different methods, and the reference DEM, where (**a**–**c**) are the altimetric profiles of experimental results corresponding to Site A–C. The profile position is at the red dashed line in Figure 10g–i.

To verify the effectiveness of the proposed method, experiments were performed using three test sites. In order to illustrate the necessity of unreliable DSM areas segmentation and hierarchical adaptive surface fitting, the methods compared in this paper were the original surface fitting, CMRF + surface fitting, and CMRF + hierarchical adaptive surface fitting. The experimental results are shown in Figure 10, and the comparison of altimetric profiles are shown in Figure 11.

Figure 10g–i show the DEM reconstruction results of the surface fitting method and the corresponding altimetric profiles are shown as a red line in Figure 11. It can be seen that the original surface fitting method had incorrect extreme values shown in the black rectangle, and the buildings were not completely filtered. This is because the unreliable DSM areas were not segmented in advance. Therefore some points of these areas were selected as ground points, and these points may be the extremely low points, or the higher points due to improper selection of grid size, thus causing the deviations in the interpolation result using ground points.

The results of the CMRF + surface fitting method are shown in Figure 10j–l, and the corresponding altimetric profiles are shown as a yellow line in Figure 11. Since the unreliable DSM areas were segmented first, and the lowest points in the grids were prevented from falling into these areas, the reconstruction results had fewer extreme values and the buildings were removed more thoroughly compared to surface fitting-based method, but there were still some buildings that had not been removed.

The results of the proposed method in this paper are shown in Figure 10m–o, and the corresponding altimetric profiles are shown as a purple line in Figure 11. It was observed that the buildings had been completely filtered out and details of the undulating terrain had been retained. The reason is that the proposed method can gradually filter out the buildings and retain most of the ground points by keeping the grid size gradually smaller and setting the adaptive threshold, achieving the fine DEM reconstruction. Comparing the reconstruction results with the reference DEM of LiDAR in Figure 10d–f, the results of the proposed method are more accurate than other approaches. It confirms that the proposed method improves the performance of an InSAR DEM reconstruction.

### 3.4. Quantitative Evaluation

In order to quantify the performance of the proposed method, the three test sites were evaluated for accuracy, and the experimental methods including surface fitting, CMRF + surface fitting, and the methods proposed in this paper were used for evaluation. The reconstructed elevation was compared with the reference elevation to obtain the absolute elevation difference of each point, and finally the statistical values were calculated as the quantitative evaluation metrics of the DEM reconstruction result, such as the maximum difference (Max), the minimum difference (Min), and the root mean square error (RMSE) of the difference which can be expressed by Equation (24):

(24) $$RMSE=\sqrt{\frac{\sum_{i=1}^{n}{\left(H_{i}-Z_{i}\right)}^{2}}{n}}$$

where $H_{i}$ is the elevation of reconstructed DEM for pixel i; $Z_{i}$ is the corresponding elevation of reference DEM; and n is the number of the pixels involved in the calculation. The comparison results are shown in Table 2.

For the surface fitting algorithm, as shown in Figure 11, the maximum difference between the red line and the green line was the evaluation index Max, and the maximal Max in the three test sites was up to 19.42 m and the maximum RMSE was 5.04 m. This is because the unreliable DSM areas were not filtered in advance, which lead to the wrong selection of the extreme points as the ground points, as shown in the lowest point of the red line in Figure 11 and the gap between the reconstruction result and the real result is relatively large. In the CMRF + surface fitting, as shown by the yellow line in Figure 11, the Max was significantly reduced, showing the necessity of CMRF segmentation, while root mean square error (RMSE) was further reduced, and the reconstruction performance was improved. Compared with the above two methods, as shown in the comparison between the purple line and the green line in Figure 11, the CMRF + hierarchical adaptive surface fitting method proposed in this paper has obvious advantages in both indicators. The RMSEs of each test site were about 1 m and the Maxs were between 1 m and 2 m. The performance was significantly improved, which confirms the effectiveness of the proposed method.

## 4. Conclusions

In this paper, we proposed a new InSAR DEM reconstruction method in order to accurately extract a DEM from DSM generated by an InSAR system. The unreliable DSM areas were segmented in advance at the selection of ground point. Experiments show that the improved CMRF segmentation method was more accurate than the MRF method. Then, the hierarchical adaptive surface fitting can be used to mark ground points and non-ground points step-by-step, making the reconstruction result more accurate. The comparison results proved the superiority of the proposed algorithm qualitatively and quantitatively. However, there is still room for improvement. On the one hand, the hierarchical adaptive surface fitting can consider more interferometric phase information. On the other hand, the acceleration of the interpolation calculation may need further research.

## Figures and Tables

**Table 1:** Estimations of Fisher distribution.

Class	M	L	μ
Layover	10.15	2.05	21.52
Shadow	12.31	5.21	3.72
Background	16.03	3.59	8.17

**Table 2:** Accuracy evaluation result.

Method	Min/m			Max/m			Root Mean Square Error (RMSE)/m		
	Site A	Site B	Site C	Site A	Site B	Site C	Site A	Site B	Site C
surface fitting	0.98	1.23	1.64	19.42	15.58	12.6	4.87	5.04	3.98
Coherent Markov Random Field (CMRF)++surface	0.81	1.01	1.33	3.12	3.79	5.18	2.32	2.76	2.84
the proposed	0.62	0.87	0.67	2.08	1.3	1.03	1.09	0.95	0.97
